# Music Memory Following Short-term Practice and Its Relationship with the Sight-reading Abilities of Professional Pianists

**DOI:** 10.3389/fpsyg.2016.00645

**Published:** 2016-05-10

**Authors:** Eriko Aiba, Toshie Matsui

**Affiliations:** ^1^Graduate School of Informatics and Engineering, University of Electro-CommunicationsTokyo, Japan; ^2^Graduate School of Systems Engineering, Wakayama UniversityWakayama, Japan

**Keywords:** expertise, musical training, musical score, auditory memory, individual differences, mistakes

## Abstract

This study investigated the relationship between the ability to sight-read and the ability to memorize a score using a behavioral experiment. By measuring the amount of memorization following short-term practice, we examined whether better sight-readers not only estimate forthcoming notes but also memorize musical structures and phrases with more practice. Eleven pianists performed the music first by sight-reading. After a 20-minute practice, the participants were asked to perform from memory without any advance notice. The number of mistakes was used as an index of performance. There were no correlations in the numbers of mistakes between sight-reading and memory trial performance. Some pianists memorized almost the entire score, while others hardly remembered it despite demonstrating almost completely accurate performance just before memory trial performance. However, judging from the participants’ responses to a questionnaire regarding their practice strategies, we found auditory memory was helpful for memorizing music following short-term practice.

## Introduction

Professional musicians are required to not only give a wonderful performance, but also to prepare thoroughly for that performance with as little practice time as possible. Pianists, especially, have many opportunities to quickly learn large numbers of new accompaniment pieces, because soloists often ask them to accompany their concert performances after they begin their training. Furthermore, pianists often support soloists while they are training. This means pianists have to finish their preparations within a much shorter time than do soloists. In addition, pianists have to read more notes simultaneously than do other instrumental performers.

Under such circumstances, sight-reading is one of the most important abilities for professional pianists. An experiment measuring the sight-reading abilities of university students studying piano performance showed a positive correlation between their ability to sight-read (performance in the first trial) and the accompanying score (performance in the fourth trial; Lehmann and Ericsson, 1996).

Many previous studies on sight-reading measured eye movements while the pianists were sight-reading or while they read the score as they played (e.g., Goolsby, 1994; Waters et al., 1997; Drai-Zerbib et al., 2012; Rosemann et al., 2015). Goolsby (1994) reported that better sight-readers did not fixate on all the notes, but crossed lines and phrase boundaries, while less proficient sight-readers tended to fixate on individual notes. Research on the eye-hand span for single melodies found that better sight-readers had a six- or seven-note span, while less proficient sight-readers only had a three- or four-note span (Sloboda, 1984).

However, the eye-hand span is affected by playing tempo and music complexity (Sloboda, 1984; Rosemann et al., 2015). In addition, pianists estimate forthcoming notes by using their knowledge of common phrases and chord progression. It is possible they grasp the subsequent notes better than those they are actually seeing. In fact, when pianists sight-read a simple tonal piano piece including several altered notes, they unintentionally corrected the altered notes to match their expectations (Sloboda, 1976). Even when the pianists were unexpectedly required to recall the score with a fill-in-the-blanks task just after sight-reading, they were able to successfully complete it (Lehmann and Ericsson, 1996). In this fill-in-the-blanks task, the pianists also estimated the notes missing from just before and after these missing notes. As a result, the pianists could fill in more blanks in the second trial. Furthermore, the authors reported a positive correlation between fill-in-the-blanks task performance and sight-reading ability.

This positive correlation may indicate better sight-readers are better able to memorize the score. Because music is mostly structural, and main or modified main phrases appear many times, memorizing previous phrases or chord progressions in a particular piece should help estimate forthcoming notes (Lehmann and McArthur, 2002).

On the other hand, in finishing short-term preparations for a performance, it is possible that reading and estimating the forthcoming notes occurs prior to score memorization. In this case, the pianists may transform the score into a performance in a partly reflexive manner, rather than memorizing it. In fact, our previous questionnaire survey of 65 pianists indicated that those who are good at sight-reading are not always good at memorizing music (Matsui and Aiba, 2015).

In this study, we hypothesized that pianists who are good at sight-reading prioritize reading the music and estimating forthcoming notes; therefore, they do not memorize the music as much. In this case, to improve their sight-reading abilities, the pianists need to strengthen the connection between the visual information (the score) and their motion (performance). This means they have to transform the score into a performance in a reflexive manner. They also need to improve their abilities to estimate the forthcoming notes. On the contrary, if memorizing music plays an important role in improving sight-reading, the pianists have to consciously memorize the music as they sight-read. The relationship between sight-reading and score memorization abilities was investigated using a behavioral experiment. We examined whether the better sight-readers not only estimated the forthcoming notes but also memorized the musical structure and phrases with practice. Previous studies concerning music memorization have been mainly conducted by interviewing musicians (e.g., Hallam, 1997; Aiello, 2001). However, in behavioral experiments by Williamon and Valentine (2002), the pianists were required to consciously memorize the score and were observed regarding differences in memorization strategies depending on the levels of the skills they attained during the training process. In our experiment, the pianists were required to play the music by sight-reading. After that, they were unexpectedly asked to perform without the score or any cue. Thus, we investigated the amount of music memorized under conditions that were as close as possible to those under which the participants usually practiced.

## Materials and Methods

## Participants

The participants were 11 professional pianists (nine females and two males). Their ages ranged from 22 to 46 years (mean ±*SD* = 28.4 ± 6.9). Participants’ piano training and performance experience ranged from 16 to 41 years (mean ±*SD* = 23.5 ± 7.3). All had graduated with a degree in piano performance from either the School of Music or the University of Arts. Eight had experienced winning more than one prize in either a domestic or international competition, and one pianist was a composer.

This experiment was approved by the University of Electro Communications Institutional Review Board for Human Subjects Research and was in accordance with the ethical standards stated in the Declaration of Helsinki. We obtained written informed consent from all the participants, and they were paid 13,000 Japanese Yen (about 100 US dollars) for their participation.

### Apparatus

The participants played a hybrid piano (AvantGrand N2, YAMAHA) in a soundproof room (Science NASAL, KAWAI). This hybrid piano generates sounds electronically but has the same mechanical key action as an acoustic grand piano. The sound signal was recorded from the hybrid piano to a note PC (X240, Lenovo) through an audio interface (UA-1010, Roland) in a Waveform Audio File Format (WAV) (16 bit, 48 kHz sampling rate) and Musical Instrument Digital Interface (MIDI). The musical score was printed on two sheets of A4 paper and placed on a music stand attached to the hybrid piano.

### Task Music

Three pieces of music were prepared (**Table 1**). Excerpts from *Variation on a Slovakian Folksong*, Op. 51 No. 3 by Dmitri B. Kabalevsky (Zen-On Music Company Limited, 1996) and *Tarentelle Brilliante*, Op. 8 by Smith (1867) were prepared as the training pieces to help the participants understand the experimental procedure. The first part of ‘Mazurka’ from *Escenas Romanticas* by Enrique Granados (Granados, 2013) was chosen as the task music. The participants practiced and performed the two training pieces using the exact same procedure as was used for the task music (Mazurka), with the exception of the memory trial. These two training pieces were easier to play than was the task music.

**Table 1:** Music used in this experiment.

Order	Composer	Title	Bars	Tempo (BPM)	Rehearsal time (minutes)
1	Kabalevsky	*Variation on a Slovakian Folksong*, Op.51 No.3	1–16	80	2
2	Smith	*Tarentelle Brilliante*, Op.8	27–58	120	5 + 5
3	Granados	*Escenas Romanticas*, Mazurka	1–45	100	10 + 10


*BPM, beats per minute*

**FIGURE 1:**
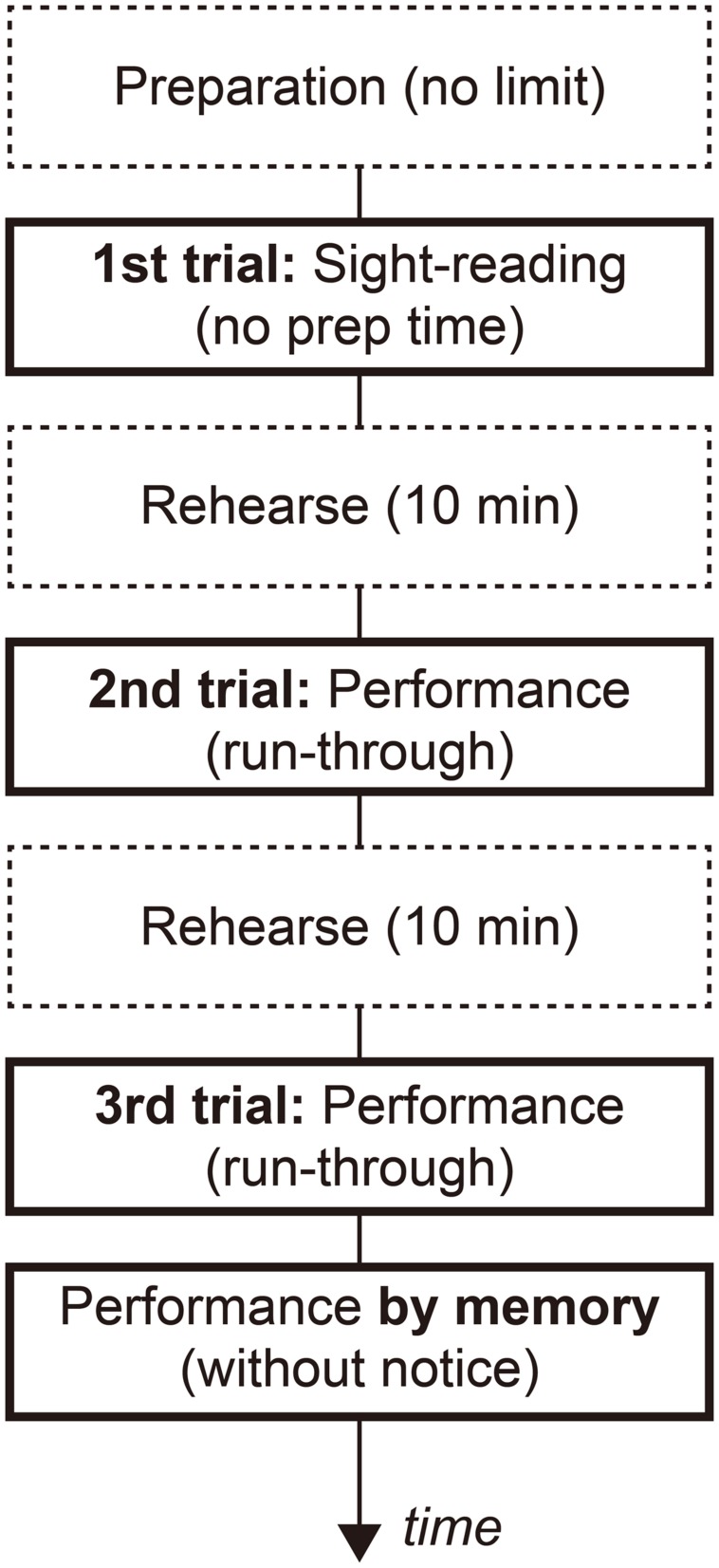
**Flow of the experimental procedure for the task music (Granados).** The vertical arrow indicates the flow of time. The boxes surrounded by a solid line indicate the trials that were included in the analysis. The participants could play with the score, with the exception of the memory trial performance. In the trials for the two training pieces, the rehearsal time was either cut or was shortened (**Table 1**), and the performance by memory trial was excluded.

The latter part and the first ending bracket were removed to shorten the length of the music; consequently, it took about a minute and a half for the participants to perform the pieces in the target tempo. The Mazurka was composed in a Romantic school style, but it also includes the essence of the composer’s unique Spanish style. Some participants (including the composer) said this Mazurka felt more familiar to them than either the Early Modern or Modern school style music, but it was more difficult to predict a chord progression in this piece than was the case with the Classical music. The difficulty level of performing this Mazurka was such that professional pianists should be able to perform it with certainty without mistakes after a 20-min practice. After the experiment, all the pianists told us they had neither played nor heard this Mazurka before. **Figure 1** shows the experimental procedure.

### Procedure

The participants’ musical histories were collected via a questionnaire before the experiment. First, the participants were given as much preparation time as they wanted to acquire the feel of the hybrid piano. They also adjusted the sound volume as they felt appropriate. At the beginning of the first trial, the musical score was placed on the stand with its back facing the participants, and the target tempo was presented to the participants using a metronome. The participants were also instructed to not stop playing until the end of the music, if possible.

In the first trial, the participants scanned through the musical score at the experimenter’s cue, and then they started to perform the task music by sight-reading. The participants were therefore given only a short time to check the measure and key of the music, and then they had to start performing within several seconds after the cue. During the rehearsal time, the participants were allowed to practice as they liked. As the rehearsal time was set based on the difficulty of the music, the easier music had a shorter rehearsal time than did the more difficult music. In the second and third trials, the participants were required to play the music from beginning to the end with the musical score. In the fourth trial, the participants were asked to perform the task music (Granados) from memory without any advance notice.

### Analysis

The recorded performance in MIDI format was transcribed to the musical score using the Digital Audio Workstation software (DAW; SONAR X3 Producer, Cakewalk). The resolution of quantization was a sixteenth note. The generated musical score and the original score were compared, and the mistakes were counted by a professional pianist, this study’s second author, who has won more than one domestic piano competition prize and has given several recitals. The mistakes were categorized into four groups: absent note, extra note, failure note, and time-shifted note (**Table 2**). For the last trial, the performance by memory, she compared which bar of the generated score was most similar to that of the original score. If it was difficult to judge how a note corresponded to the bar, that note was excluded from the analysis. The notes of a bar that were not performed were all treated as absent notes. The melody was mainly used as a criterion for judgment. Additionally, the rhythm and the harmony were checked. The average tempo was calculated from the entire duration of each performance (**Table 3**).

**Table 2:** Mistake types and their definitions.

Mistake type	Definition
Absent note	A note that was not played, although it is in the original score.
Extra note	A note that was played, although it is not in the original score.
Failure note	A note close to the original note was played by mistake.
Time-shifted note	A note was played more than one beat earlier or later than the original note.


**Table 3:** Calculated average tempi for each participant in each trial (BPM).

Participant ID	1st trial	2nd trial	3rd trial
S01M03	114	115	
S02M05	64	83	90
S03M02	75	107	107
S04M08	88	83	86
S05M01	81	82	82
S06M10	85	82	92
S07M07	84	84	84
S08M09	90	94	91
S09M11	79	79	83
S10M04	97	89	97
S11M06	151	100	100
Mean	91.5	90.6	91.2
*SD*	22.3	11.2	7.6


*BPM = beats per minute.*

## Results

### Sight-Reading

**FIGURE 2:**
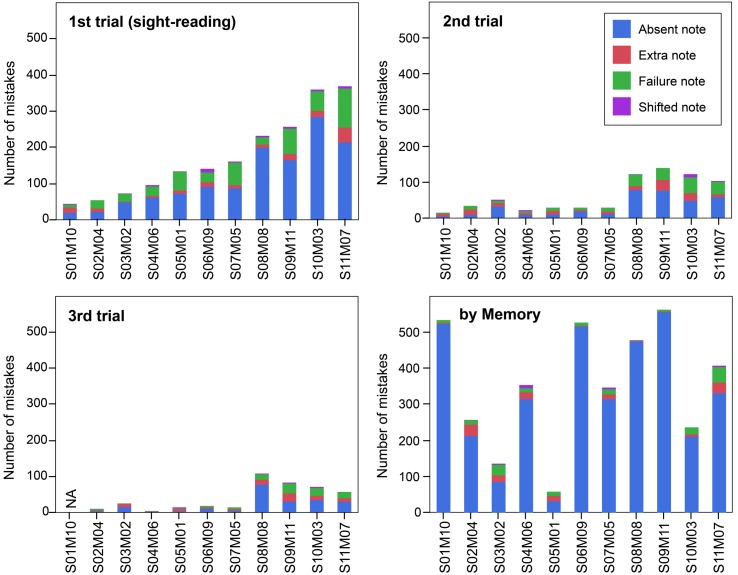
**Each participant’s number of mistakes for each trial in which they played the task music (Granados).** Vertical bars indicate each participant’s number of mistakes, with the bars arranged according to the number of mistakes in the sight-reading trial. The participant ID was created based on their sight-reading (from S01 to S11) and their music memorization (from M01 to M11) scores. For example, Participant S01M03 made the fewest mistakes in the sight-reading trial and the third fewest mistakes in memory performance. The color indicates the type of mistake made. There were 581 total notes in the task music.

**Figure 2** shows the average number of mistakes for each participant in each trial in which the participants played the task music (Granados). The mistakes in the sight-reading trial ranged from 44 to 369 notes. The best sight-reader, S01M10, was a composer, and the calculated average tempo was about 114 beats per minute (BPM). The best sight-reader’s mistakes accounted for fewer than 8% of the total notes. He did not perform the third trial, since he claimed that even if he could practice for 10 more minutes, the number of mistakes he made would not decrease. On the other hand, S11M07 had the highest number of mistakes. She explained this as being a result of her misunderstanding the unit of the beat. In fact, S11M07’s calculated average tempo was about 151 BPM.

In the sight-reading trial, mistakes consisted mainly of absent notes. It was therefore observed that the melody and bass parts were played continuously, while the parts with large leaps and quick passages were cut, and all performances maintained the original impression of the task music.

The correlations between the number of sight-reading mistakes and participants’ profiles were calculated by single regression analysis. All the statistical analyses in this study were computed using statistical software (JMP9.0.3, SAS Institute Inc.). There were no significant correlations between the number of mistakes during sight-reading and profile parameters, such as age (*R*^2^ = 0.14), training onset age (*R*^2^ = 0.00), or training period (*R*^2^ = 0.13).

### Performance with the Musical Score

The number of mistakes decreased between the second and third trials for all participants. In the third trial, the performance practice seemed to be completed. The correlations between the number of mistakes in the first and second trials, and between those of the first and third trials were also calculated using single-regression analysis. Relatively high correlations were observed in both comparisons (1st and 2nd: *R*^2^ = 0.64; and 1st and 3rd: *R*^2^ = 0.51).

### Relationship between Sight-Reading and Music Memorization

As shown in **Figure 2**, there was no correlation in the number of mistakes between the sight-reading trial and the performance in the memory trial (*R*^2^ = 0.02). After excluding participants S01M10 and S11M07, because S01M10 did not perform the third trial and S11M07 misunderstood the unit of the beat in the first trial, the correlation still did not reach a level of significance (*R*^2^ = 0.09). This implies there are large individual differences in the memory strategies pianists use while playing.

### Relationship between Amount and Strategy of Memorization

In the questionnaire, the participants were asked about how they practice when they start learning new music. The choices were as follows: A, Read the score; B, Analyze the structure of the music; C, Practice until you can play the entire score; D, Listen to other pianists’ CDs; E, Memorize the score (visually); F, Memorize as sound (auditory); G, Memorize as action (motion; e.g., fingering); H, Record own performance and listen; and I, Other. **Table 4** shows participants’ responses of choices E, F, and G, which are related to music memorization.

**Table 4:** Each participant’s memorization strategy response.

Participant ID	E, Memorize as score	F, Memorize as sound	G, Memorize as motion
S05M01	No	Yes	Yes
S03M02	No	Yes	Yes
S10M03	Yes	Yes	Yes
S02M04	Yes	Yes	No
S07M05	Yes	Yes	Yes
S04M06	Yes	Yes	No
S11M07	Yes	Yes	Yes
S08M08	No	No	Yes
S06M09	Yes	Yes	Yes
S01M10	No	No	Yes
S09M11	No	No	No


Furthermore, independent-sample two-tailed *t*-tests were performed to examine the number of mistakes in the memory trial performances for each type of memorization strategy. For Choice F, the number of mistakes was compared between the eight participants who answered ‘yes’ and the three who answered ‘no’. As a result, participants who answered that memorizing from sound was one of their strategies had significantly fewer mistakes in the memory trial performance (*t* = 2.58, df = 9, and *p* < 0.05). For Choice E, the number of mistakes was compared between the six participants who answered ‘yes’ and the five who answered ‘no’. There were no significant differences in number of mistakes on the memory trial (*t* = -0.01, df = 9, and n.s.). For Choice G, the number of mistakes was compared between the three participants who answered ‘yes’ and the eight who answered ‘no’. In addition, there were no significant differences in number of mistakes on the memory trial (memorize as action; *t* = 0.43, df = 9, and n.s.). Although the number of participants was limited, it seemed that at least Choice F (memorize as sound) appeared to be more effective than the other two strategies.

### Memorized Part of Music

**FIGURE 3:**
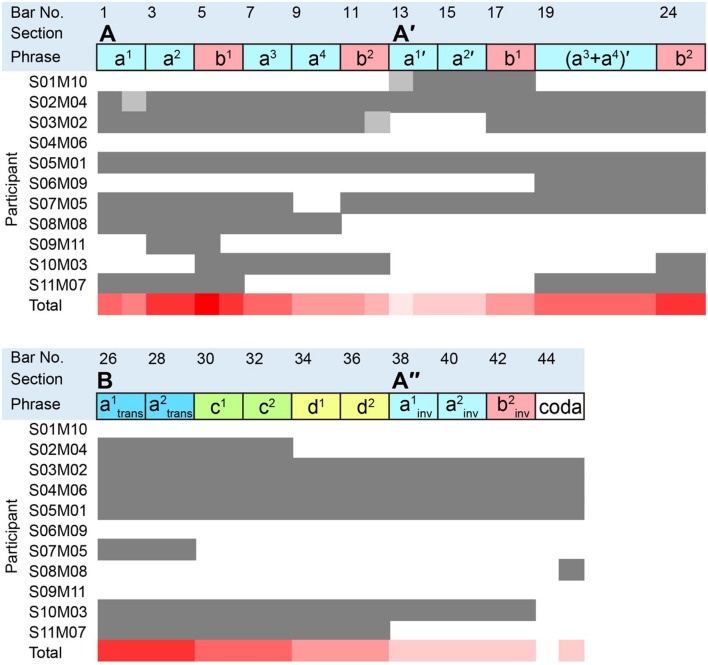
**Structure of the task music (Granados) and bars memorized by each participant.** Capital letters indicate the section of the music, and lower-case letters indicate the phrase types. The superscript symbol for each lower-case letter indicates the developed phrases from each type of phrase. The apostrophe indicates the variation of each type of section or phrase. The subscripts ‘trans’ and ‘inv’ represent the abbreviations ‘transposition’ and ‘inversion’, respectively. The filled squares show the bars memorized by each participant. The dark gray square indicates bars for which the participant memorized both right and left hand notes (including bars with whole remaining notes in either the right or left hand). The light gray square indicates bars for which the participant memorized only the right or left hand.

To illustrate the performance details, the structure of the task music by Granados is presented in **Figure 3.** This musical analysis was conducted by the same pianist who counted the number of mistakes.

**FIGURE 4:**
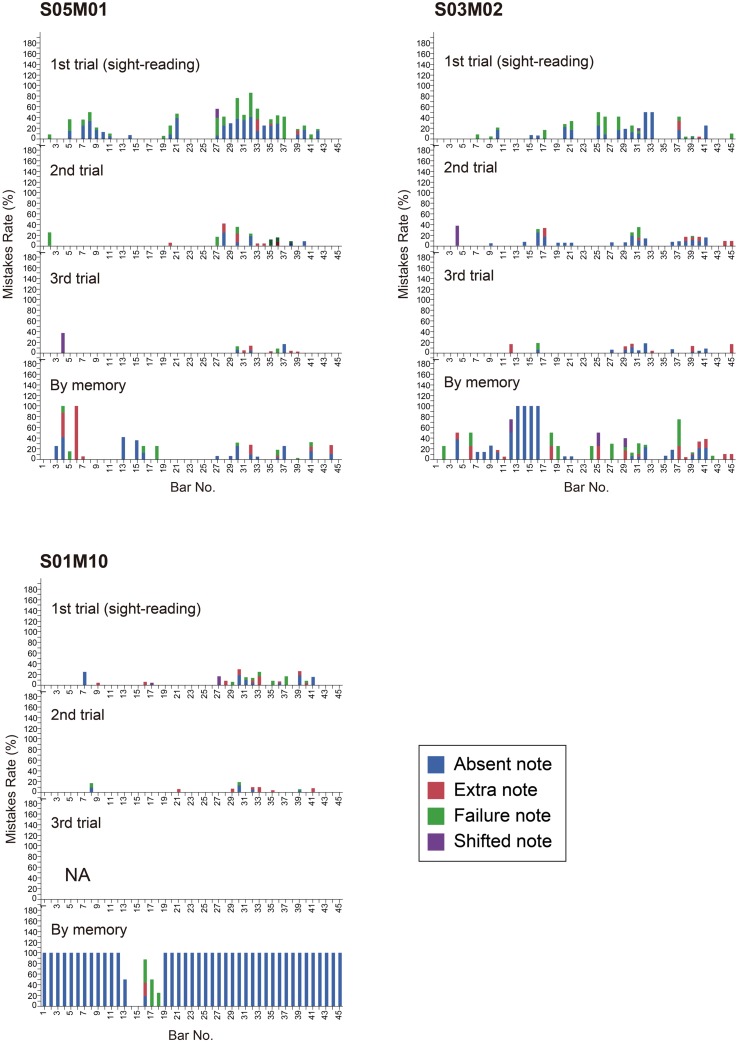
**Mistake rate by bar number for Participants S05M01, S03M02, and S01M10 in each trial they played the task music (Granados).** The color indicates the type of mistake. Each panel represents one trial.

Surprisingly, S05M01 and S03M02 memorized almost the entire score in spite of having only a 20-min practice (see **Figures 3** and **4** for greater detail). They memorized both the structure and the phrase of the task music. As S03M02 did not seem to remember the music from Bar No. 13 to Bar No. 16, she just played the music from Bar No. 26 to Bar No. 29 as an alternative. Bar No. 13 to Bar No. 16 corresponds to Phrases a1 and a2, and Bar No. 26 to Bar No. 29 corresponds to Phrases a1trans and a2trans. After S03M02 skipped Section A once, she apparently realized her mistake of skipping Bar No. 13 to Bar No. 16 in the middle of playing. These two participants showed relatively good sight-reading performance.

On the other hand, S01M10 only memorized the music from Bar No. 13 to Bar No. 18, in spite of the fact he showed the best performance in the sight-reading trial. As Bar No. 13 to Bar No. 18 corresponds to Phrases a1’, a2’, and b1, it seems that S01M10 just memorized the components of the main phrase. Since S01M10 only had a 10-min practice, his memorization capacity would naturally be affected. However, in the questionnaire he responded that he usually does not memorize a score, which could be attributed to the fact that S01M10 is a composer who has a remarkably good ability to estimate the preceding notes.

**FIGURE 5:**
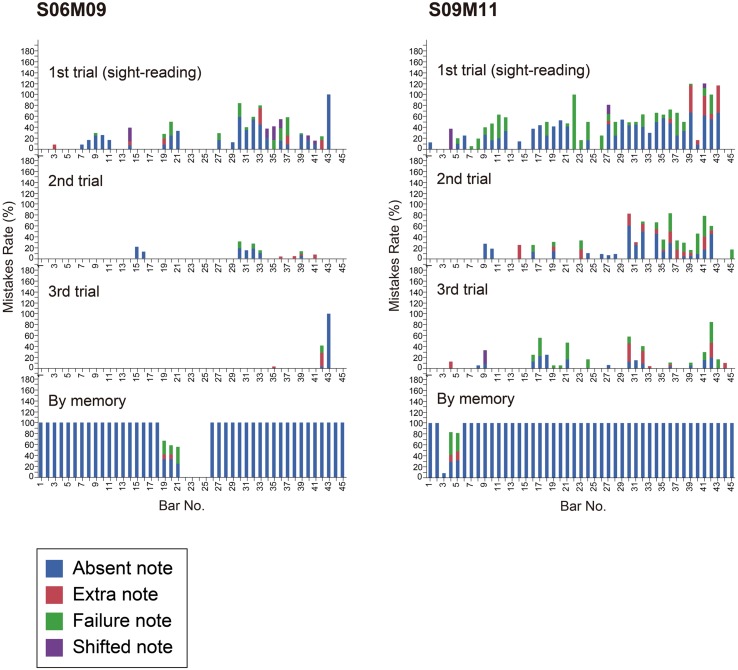
**Mistake rate by bar number for Participants S06M09 and S09M11 in each trial they played the task music (Granados).** The color indicates the type of mistake. Each panel represents one trial.

**FIGURE 6:**
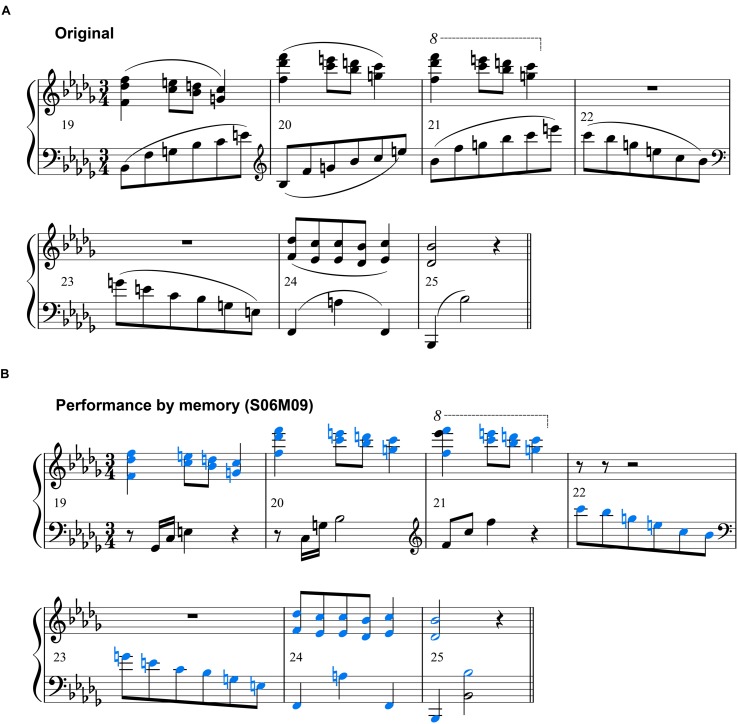
**(A)** Excerpt from the original score (numbers indicate the bar number). **(B)** The score that was generated from S06M09’s memory performance of the same bar number. Blue-colored notes indicate notes played correctly. These scores were generated by music notation software (MuseScore 2.0.2, MuseScore).

**FIGURE 7:**
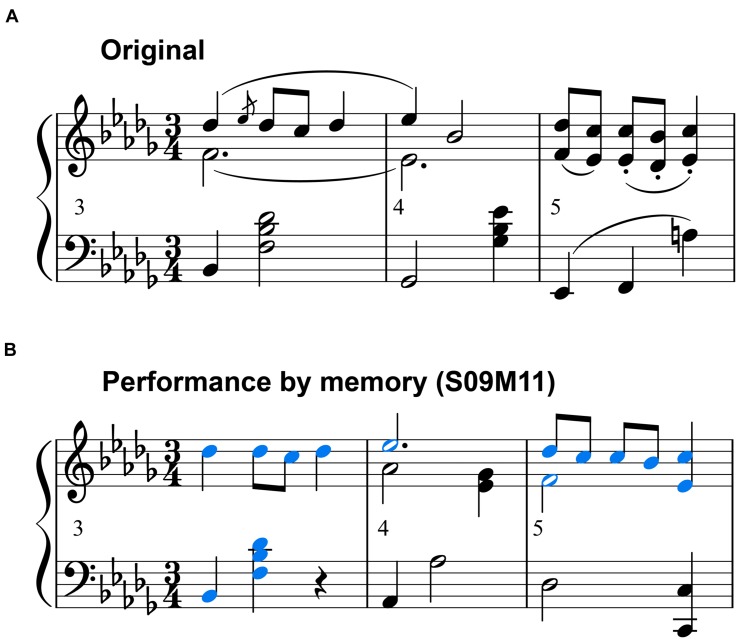
**(A)** Excerpt from the original score (numbers indicate the bar number). **(B)** Score that was generated from S09M11’s memory performance of the same bar number. Blue-colored notes indicate notes played correctly. These scores were generated by music notation software (MuseScore 2.0.2, MuseScore).

In addition, S06M09 and S09M11 only memorized several phrases or their components (see **Figures 3** and **5** for detail). S06M09 memorized the music from Bar No. 19 to Bar No. 25, which corresponds to Phrases (a3+a4)’ and b2. S09M11 memorized the music from Bar No. 3 to Bar No. 5, which corresponds to Phrase a2 and the first part of b1. **Figures 6B** and **7B** show excerpts of the score generated from the memory performances of S06M09 and S09M11, respectively. This comparison to the original scores (**Figures 6A** and **7A**) suggests they remembered the impressive components of the phrases, although they did not memorize the musical structure or entire sections of the music.

S08M08 memorized almost the entire Section A; however, Section A’ was not played in spite of the fact it was quite similar to Section A.

Although S10M03 and S11M07 memorized almost the entire Section A, neither played Section A’. However, in addition to Section A, both participants played Section B and part of Section A”, which suggests that they memorized almost all types of phrases, although their memory of the musical structure was ambiguous, and they could memorize entire sections of music.

S02M04 and S07M05 memorized the music from Bar No. 1 to Bar No. 33 and from Bar No. 1 to Bar No. 29, which represents the earlier parts of the music. S02M04 played a mixture of Phrases a2 and a2’ as part of Phrase a2, and S07M05 played a mixture of Phrases a1 and a1’ as part of Phrase a1. This suggests that the reason why they could play Sections A and A’ is that they memorized the musical structure and the music from an entire section.

In contrast to these participants, S04M06 memorized Sections B and A”, which comprise the latter part of the music. Thus, S04M06 might have only remembered the latter part of the music. While the memorized sections differed between S02M04 and S07M05, S04M06 also memorized the music from an entire section. These results indicate differences in whether the participants memorized the musical structure; however, many participants memorized the music from smaller units of a section.

## Discussion

Sight-reading abilities initially showed large individual differences. After only a 20-min practice, all the participants were able to play the task music, and the individual differences decreased. There were significant correlations between the number of mistakes in the first and second trials, and between the first and third trials. This corresponds to Lehmann and Ericsson’s (1996) result that the ability to sight-read is consistent with final performance. The best sight-reader was the composer. This result was also consistent with Lehmann and Ericsson’s (1996) study finding that the ability to estimate forthcoming notes helps sight-reading.

The absence of a significant correlation in the number of mistakes between the sight-reading trial and memory performance suggests that memorizing previous phrases may not necessarily help in the estimation of forthcoming music notes. Some good sight-readers may transform the musical score into their performance in a partly reflexive manner instead of memorizing it. However, this also suggests that their memory based on the estimation of forthcoming notes during sight-reading is not very substantive. It is possible that prior knowledge of common phrases and chord progressions has a greater effect on sight-reading ability than does the effect of temporary memory.

On the other hand, it was surprising to learn that S05M01 and S03M02 memorized almost the entire musical score. Their usual memorization strategy (**Table 4**) was a combination of sound and motion. This suggests they are not dependent on the musical scores in their regular practice. For these two participants, performance practice may mean music memorization. As Aiello and Williamon (2002) described, some methods of memorizing piano music emphasize auditory memory, and visual memory is more important than kinesthetic memory. Moreover, Aiello (2001) reported that inexperienced musicians rely on kinesthetic memory. Concert pianists may only depend on kinesthetic memory when they play fast virtuoso passages, because this kind of passage is too difficult to remember if they use other types of memory (Aiello and Williamon, 2002). Although the number of participants was limited in the current study, the results suggest that kinesthetic memory could aid music memorization within a short-term practice.

The participants who answered that memorizing by sound was one of their most important memorization strategies also showed good memory. This suggests auditory memory is helpful in music memorization following short-term practice. On the other hand, visual memory might be helpful for maintaining music memory and for memorizing the grand scale of the music.

According to previous studies (e.g., Gruson, 1988; Aiello, 2001; Aiello and Williamon, 2002), most professional pianists report that analyzing musical structure is helpful for music memorization. In the current experiment, the participants were unexpectedly required to perform the music from memory after only a 20-min practice. Twenty minutes does not seem to be adequate time to memorize musical structure. In addition, as the musical analysis is normally performed on the musical score, the musicians write its structure on the score. Although this is just a hypothesis, they could have memorized the musical structure as a type of visual memory.

Most participants memorized a unit of a section of music, which took about 25 s for them to play. It seems this duration of musical unit was important for the musicians’ music memorization. If they recalled the music within this length of musical unit, the musical structure could help them recall which section they should play and in which order.

### Limitations

As we mentioned in Section “Task Music”, the task music by Granados was composed in the Romantic school style but it also included the composer’s unique Spanish style. Japanese pianists are not as familiar with Spanish-style music as they are with the music from other Romantic composers. In our questionnaire, more or less, no participant answered that he/she was good at performing Spanish composers’ music. This might indicate we have some new findings concerning music familiarity and the difficulty of estimating chord progression (e.g., tonal vs. atonal music). As the index of performance for each trial, we adopted the number of mistakes. However, as most mistakes were mainly absent notes, the number of mistakes did not directly correspond to the impressions of performance quality. Instead of at the note level, we might better evaluate performance using the phrase or motif levels of indices. Tempo and expression also affect impressions of performance quality. The listeners’ impression evaluations could also be used as an index of performance.

## Conclusion

In this study, the relationship between sight-reading and score memorization abilities was investigated using a behavioral experiment. By measuring the amount of memorization following short-term practice, we examined whether the better sight-readers not only estimated forthcoming notes but also memorized the musical structure and phrases with practice.

As a result, there was no correlation in the number of performance mistakes occurring in the sight-reading and memory trials. Large individual differences were found in the memory strategies used while playing. However, judging from the results, which were based on the participants’ responses to the questionnaire concerning their practice strategies, we found that auditory memory was helpful for memorizing music following short-term practice.

## Author Contributions

Both authors planned this research. EA ran the experiment, analyzed the experimental data and drafted most part of the manuscript. She received grants from JSPS for this topic. TM collected the questionnaire, analyzed the experimental data and drew the figures for this manuscript. She received grants from Yamaha Music Foundation.

## Conflict of Interest Statement

The authors declare that the research was conducted in the absence of any commercial or financial relationships that could be construed as a potential conflict of interest.
